# Supplementary material for: Efficacy of Deferoxamine Mesylate in Serum and Serum-Free Media: Adult Ventral Root Schwann Cell Survival Following Hydrogen Peroxide-Induced Cell Death
Source: Cells. 2025 Mar 20;14(6):461. doi: 10.3390/cells14060461 (PMC11940984; doi:10.3390/cells14060461)
Supplement: Supplementary file 1 [file cells-14-00461-s001.zip › cells-3488771-supplementary.pdf]

Table S1. Media components

Media Abbreviations	Ingredients
D10s	90% DMEM (Dulbecco's Modified Eagle Medium, Gibco), 10% HIFBS (Heat Inactivated Fetal Bovine Serum, Gibco, Aliquoted then heated in 56°C water bath for 30 minutes), Gentamicin (10mg/mL, Gibco), Glutamax (200mM, Gibco)
CDM	100% DMEM/F12 (Dulbecco's Modified Eagle Medium/Nutrient Mixture F-12), Glutamax (200mM, Gibco), Gentamicin (50mg/mL), Bovine Insulin (10µg/mL, Millipore Sigma I6634-100MG), Human Transferrin (10µg/mL, Millipore Sigma T8158-1G), Putrescine dihydrochloride (200µM, Millipore Sigma P5780-5G), Sodium Selenite (30nM, Millipore Sigma S5261-25G)
____ +Mit	Base media plus: Forskolin (2mM, Sigma Aldrich), BPEX (Bovine Pituitary Extract, 20mg/mL)
____ +3F	Base media plus: Forskolin (2mM, Sigma Aldrich), Neuregulin Beta 1 (10ng/mL), BPEX (Bovine Pituitary Extract, 20mg/mL)
____ +DFO	Base media (+/- Mit or +/- 3F) plus: 200µM Deferoxamine Mesylate (Sigma Aldrich D9533)
____ +FGF2	Base media (+/- Mit or +/- 3F) plus: 10ng/mL Fibroblast Growth Factor 2 (PeproTech)
____ +FGF5	Base media (+/- Mit or +/- 3F) plus: 10ng/mL Fibroblast Growth Factor 5 (PeproTech)

Table S2. Pretreatment Groups for Survival Quantification (Fig. 2, 3, 4)

Serum Containing (Fig. 3)	Serum-Free (Fig. 4)	Growth Factors (Fig. 2)
CDM -H_2O_2 (n=12)	D10S -H_2O_2 (n=12)	D10S Mit -H_2O_2 (n=48)
CDM +H_2O_2 (n=16)	D10S +H_2O_2 (n=16)	D10S Mit +H_2O_2 (n=16)
CDM DFO -H_2O_2 (n=12)	D10S DFO -H_2O_2 (n=12)	D10S 3F -H_2O_2 (n=48)
CDM DFO +H_2O_2 (n=16)	D10S DFO +H_2O_2 (n=16)	D10s 3F +H_2O_2 (n=16)
CDM 3F -H_2O_2(n=12)	D10S 3F -H_2O_2 (n=12)	D10S Mit FGF2 -H_2O_2 (n=48)
CDM 3F +H_2O_2 (n=16)	D10S 3F +H_2O_2 (n=16)	D10S Mit FGF2 +H_2O_2 (n=16)
CDM 3F DFO -H_2O_2 (n=12)	D10S 3F DFO -H_2O_2 (n=12)	D10S Mit FGF5 -H_2O_2 (n=48)
CDM 3F DFO +H_2O_2 (n=16)	D10S 3F DFO +H_2O_2 (n=16)	D10S Mit FGF5 +H_2O_2 (n=16)

Table S3. Pretreatment Groups for WC-CTCF (Hif1a)

Serum-Free	Serum Containing
CDM -H_2O_2 (n=4)	D10S -H_2O_2 (n=4)
CDM +H_2O_2 (n=6)	D10S +H_2O_2 (n=6)
CDM DFO -H_2O_2 (n=4)	D10S DFO -H_2O_2 (n=4)
CDM DFO +H_2O_2 (n=5)	D10S DFO +H_2O_2 (n=6)
CDM 3F -H_2O_2(n=4)	D10S 3F -H_2O_2 (n=4)
CDM 3F +H_2O_2 (n=6)	D10S 3F +H_2O_2 (n=6)
CDM 3F DFO -H_2O_2 (n=4)	D10S 3F DFO -H_2O_2 (n=4)
CDM 3F DFO +H_2O_2 (n=6)	D10S 3F DFO +H_2O_2 (n=6)

Table S4. Pretreatment Groups for WC-CTCF (Collagen IV)

Serum-Free	Serum Containing
CDM -H_2O_2 (n=5)	D10S -H_2O_2 (n=4)
CDM +H_2O_2 (n=4)	D10S +H_2O_2 (n=4)
CDM DFO -H_2O_2 (n=4)	D10S DFO -H_2O_2 (n=8)
CDM DFO +H_2O_2 (n=4)	D10S DFO +H_2O_2 (n=4)
CDM 3F -H_2O_2(n=4)	D10S 3F -H_2O_2 (n=4)
CDM 3F +H_2O_2 (n=4)	D10S 3F +H_2O_2 (n=4)
CDM 3F DFO -H_2O_2 (n=4)	D10S 3F DFO -H_2O_2 (n=4)
CDM 3F DFO +H_2O_2 (n=4)	D10S 3F DFO +H_2O_2 (n=4)

Table S5. Pretreatment Groups for RT-qPCR (Fig. 7, 8)

Serum Containing	Serum-Free
CDM -H_2O_2 (n=1)	D10S -H_2O_2 (n=1)
CDM DFO +H_2O_2 (n=1)	D10S DFO +H_2O_2 (n=1)
CDM 3F +H_2O_2 (n=1)	D10S 3F +H_2O_2 (n=1)
CDM 3F DFO +H_2O_2 (n=1)	D10S 3F DFO +H_2O_2 (n=1)

Table S6. Gene List and Associated Pathways for RT-qPCR

Anti-Apoptotic	Akt1, Bcl2, Bcl2a1 (Bfl-1, A1), Bcl2l1 (Bcl-xl), Bcl2l1 (Bcl-xl), Bcl2l1 (Bcl-xl), Birc3 (cIAP1), Casp2, Igf1r, Mcl1, Tnfrsf11b (Opg), Traf2, Xiap
Pro-Apoptotic	Abl1, Apaf1, Atp6v1g2, Bax, Bcl2l11, Birc2 (c-IAP2), Casp1 (Ice), Casp3, Casp6, Casp7, Casp9, Cd40, Cd40lg, Cflar (Casper), Cyld, Dffa, Fas, Faslg, Gadd45a, Nol3, Spata2, Sycp2, Tnf, Tnfrsf1a (Tnfr1), Tnfrsf10b (QuantiNova Symbol: AABR07018323.1), Tp53 (p53)
Necrotic	Atp6v1g2, Bmf, Commd4, Cybb, Cyld, Defb1, Dpysl4, Foxi1, Galnt5, Grb2, Hspbap1, Kcnip1, Mag, Olr1583, Parp1 (Adprt1), Parp2, Pvr, Rab25, RGD1311517, Dennd4a, Spata2, Sycp2, Maco1, Tnfrsf1a (Tnfr1), Tnfrsf4 (Ox40), Tnfrsf8, Txnl4b
Autophagy	Akt1, App, Atg12, Atg16l1, Atg3, Atg5, Atg7, Bax, Bcl2, Bcl2l1 (Bcl-xl), Bcl2l1 (Bcl-xl), Bcl2l1 (Bcl-xl), Becn1, Casp3, Ctsb, Ctss, Esr1 (Erα), Fas, Gaa, Htt, Ifng, Igf1, Ins2, Irgm, Map1lc3a, Mapk8 (Jnk1), Nfkb1, Pik3c3 (Vps34), Pten, Rps6kb1, Snca, Sqstm1, TnfTp53 (p53), Ulk1

Table S7. P values for cell survival counts in Serum Groups

D10S -H_2O_2 vs D10S +H_2O_2, p=1.157e-13, ****	D10S 3F -H_2O_2 vs D10S 3F +H_2O_2, p=3.730e-14, ****
D10S - H_2O_2 vs D10S DFO +H_2O_2 p=1.455e-01, ns	D10S 3F - H_2O_2 vs D10S DFO +H_2O_2 p=5.124e-02, ns
D10S -H_2O_2 vs D10S DFO -H_2O_2, p=7.974e-01, ns	D10S 3F -H_2O_2 vs D10S DFO -H_2O_2, p=5.484e-01, ns
D10S -H_2O_2 vs D10S 3F -H_2O_2, p=9.999e-01, ns	D10S 3F -H_2O_2 vs D10S 3F DFO -H_2O_2, p=1.000e+00, ns
D10S -H_2O_2 vs D10S 3F +H_2O_2, p=4.063e-14, ****	D10S 3F -H_2O_2 vs D10S 3F DF0 +H_2O_2, p=4.464e-03, **
D10S -H_2O_2 vs D10S 3F DFO -H_2O_2, p=1.000e+00, ns	D10S 3F + H_2O_2 vs D10S DFO +H_2O_2 p=9.259e-14, ****
D10S -H_2O_2 vs D10S 3F DF0 +H_2O_2, p=1.747e-02, *	D10S 3F +H_2O_2 vs D10S DFO -H_2O_2, p=8.771e-14, ****
D10S + H_2O_2 vs D10S DFO +H_2O_2, p=2.397e-10, ****	D10S 3F +H_2O_2 vs D10S 3F DFO -H_2O_2, p=3.797e-14, ****
D10S +H_2O_2 vs D10S DFO -H_2O_2, p=2.240e-11, ****	D10S 3F +H_2O_2 vs D10S 3F DF0 +H_2O_2, p=1.181e-13, ****
D10S +H_2O_2 vs D10S 3F -H_2O_2, p=1.039e-13, ****	D10S DFO- H_2O_2 vs D10S DFO +H_2O_2 p=9.663e-01, ns
D10S +H_2O_2 vs D10S 3F +H_2O_2, p= 9.664e-02, ns	D10S DFO -H_2O_2 vs D10S 3F DFO -H_2O_2, p=6.532e-01, ns
D10S +H_2O_2 vs D10S 3F DFO -H_2O_2, p=1.090e-13, ****	D10S DFO -H_2O_2 vs D10S 3F DF0 +H_2O_2, p=5.976e-01, ns
D10S +H_2O_2 vs D10S 3F DF0 +H_2O_2, p= 1.577e-08, ****	D10S DFO +H_2O_2 vs D10S 3F DFO +H_2O_2, p=9.893e-01, ns
D10S DFO +H2O2 vs D10S 3F DFO -H2O2, p= 7.942e-02, ns	D10S 3F DFO +H_2O_2 vs D10S 3F DFO -H_2O_2, p=7.813e-03, **

Table S8. P values for SC survival counts in Serum-free groups

CDM -H_2O_2 vs CDM +H_2O_2, p=1.128e-01, ns	CDM 3F -H_2O_2 vs CDM 3F +H_2O_2, p= 5.037e-02, ns
CDM - H_2O_2 vs CDM DFO +H_2O_2 p=1.000e+00, ns	CDM 3F - H_2O_2 vs CDM DFO +H_2O_2 p=9.995e-01, ns
CDM -H_2O_2 vs CDM DFO -H_2O_2, p=9.917e-01, ns	CDM 3F -H_2O_2 vs CDM DFO -H_2O_2, p=1.000e+00, ns
CDM -H_2O_2 vs CDM 3F -H_2O_2, p=9.951e-01, ns	CDM 3F -H_2O_2 vs CDM 3F DFO -H_2O_2, p=6.121e-01, ns
CDM -H_2O_2 vs CDM 3F +H_2O_2, p=4.214e-03, ns	CDM 3F -H_2O_2 vs CDM 3F DF0 +H_2O_2, p=9.987e-01, ns
CDM -H_2O_2 vs CDM 3F DFO -H_2O_2, p=1.813e-01, ns	CDM 3F + H_2O_2 vs CDM DFO +H_2O_2 p=4.145e-03. **
CDM -H_2O_2 vs CDM 3F DF0 +H_2O_2, p=8.513e-01, ns	CDM 3F +H_2O_2 vs CDM DFO -H_2O_2, p= 6.144e-02, ns
CDM + H_2O_2 vs CDM DFO +H_2O_2, p=1.374e-01, ns	CDM 3F +H_2O_2 vs CDM 3F DFO -H_2O_2, p=9.434e-01, ns
CDM +H_2O_2 vs CDM DFO -H_2O_2, p=5.548e-01, ns	CDM 3F +H_2O_2 vs CDM 3F DF0 +H_2O_2, p=1.396e-01, ns
CDM +H_2O_2 vs CDM 3F -H_2O_2, p=5.049e-01, ns	CDM DFO- H_2O_2 vs CDM DFO +H_2O_2 p=9.989e-01, ns
CDM +H_2O_2 vs CDM 3F +H_2O_2, p=9.281e-01, ns	CDM DFO -H_2O_2 vs CDM 3F DFO -H_2O_2, p=6.582e-01, ns
CDM +H_2O_2 vs CDM 3F DFO -H_2O_2, p=1.000e+00, ns	CDM DFO -H_2O_2 vs CDM 3F DF0 +H_2O_2, p=9.995e-01, ns
CDM +H_2O_2 vs CDM 3F DF0 +H_2O_2, p=8.185e-01, ns	CDM DFO +H_2O_2 vs CDM 3F DFO +H_2O_2, p=9.259e-01, ns
CDM DFO +H2O2 vs CDM 3F DFO -H2O2, p=2.260e-01, ns	CDM 3F DFO +H_2O_2 vs CDM 3F DFO -H_2O_2, p=8.843e-01, ns

Table S9. P values for Hif1a WC-CTCF

D10S -H_2O_2 vs D10S +H_2O_2, p= 1.800e-02	D10S 3F -H_2O_2 vs D10S 3F +H_2O_2, p= 2.300e-04
D10S - H_2O_2 vs D10S DFO +H_2O_2 p= 2.700e-02	D10S 3F - H_2O_2 vs D10S DFO +H_2O_2 p= 5.000e-03
D10S -H_2O_2 vs D10S DFO -H_2O_2, p= 2.500e-02	D10S 3F -H_2O_2 vs D10S DFO -H_2O_2, p= 3.000e-03
D10S -H_2O_2 vs D10S 3F -H_2O_2, p= 4.000e-02	D10S 3F -H_2O_2 vs D10S 3F DFO -H_2O_2, p= 2.740e-01
D10S -H_2O_2 vs D10S 3F +H_2O_2, p= 1.500e-02	D10S 3F -H_2O_2 vs D10S 3F DF0 +H_2O_2, p= 1.600e-02
D10S -H_2O_2 vs D10S 3F DFO -H_2O_2, p= 1.300e-02	D10S 3F + H_2O_2 vs D10S DFO +H_2O_2 p= 1.000e-02
D10S -H_2O_2 vs D10S 3F DF0 +H_2O_2, p= 2.900e-02	D10S 3F +H_2O_2 vs D10S DFO -H_2O_2, p= 3.000e-02
D10S + H_2O_2 vs D10S DFO +H_2O_2, p= 3.000e-03	D10S 3F +H_2O_2 vs D10S 3F DFO -H_2O_2, p= 5.960e-01
D10S +H_2O_2 vs D10S DFO -H_2O_2, p= 1.700e-02	D10S 3F +H_2O_2 vs D10S 3F DF0 +H_2O_2, p= 6.000e-03
D10S +H_2O_2 vs D10S 3F -H_2O_2, p= 7.000e-05	D10S DFO- H_2O_2 vs D10S DFO +H_2O_2 p= 5.970e-01
D10S +H_2O_2 vs D10S 3F +H_2O_2, p= 9.090e-01	D10S DFO -H_2O_2 vs D10S 3F DFO -H_2O_2, p= 1.000e+00
D10S +H_2O_2 vs D10S 3F DFO -H_2O_2, p= 8.280e-01	D10S DFO -H_2O_2 vs D10S 3F DF0 +H_2O_2, p= 6.800e-02
D10S +H_2O_2 vs D10S 3F DF0 +H_2O_2, p= 1.000e-03	D10S DFO +H_2O_2 vs D10S 3F DFO +H_2O_2, p= 7.830e-01
D10S DFO +H2O2 vs D10S 3F DFO -H2O2, p= 9.880e-01	D10S 3F DFO +H_2O_2 vs D10S 3F DFO -H_2O_2, p= 9.180e-01
CDM -H_2O_2 vs CDM +H_2O_2, p= 5.600e-01	CDM 3F -H_2O_2 vs CDM 3F +H_2O_2, p= 9.850e-01
CDM - H_2O_2 vs CDM DFO +H_2O_2 p= 9.220e-01	CDM 3F - H_2O_2 vs CDM DFO +H_2O_2 p= 4.440e-01
CDM -H_2O_2 vs CDM DFO -H_2O_2, p= 9.390e-01	CDM 3F -H_2O_2 vs CDM DFO -H_2O_2, p= 1.040e-01
CDM -H_2O_2 vs CDM 3F -H_2O_2, p= 1.000e+00	CDM 3F -H_2O_2 vs CDM 3F DFO -H_2O_2, p= 2.630e-01
CDM -H_2O_2 vs CDM 3F +H_2O_2, p= 9.890e-01	CDM 3F -H_2O_2 vs CDM 3F DF0 +H_2O_2, p= 4.800e-01
CDM -H_2O_2 vs CDM 3F DFO -H_2O_2, p= 8.100e-01	CDM 3F + H_2O_2 vs CDM DFO +H_2O_2 p= 4.240e-01
CDM -H_2O_2 vs CDM 3F DF0 +H_2O_2, p= 9.770e-01	CDM 3F +H_2O_2 vs CDM DFO -H_2O_2, p= 3.840e-01
CDM + H_2O_2 vs CDM DFO +H_2O_2, p= 9.560e-01	CDM 3F +H_2O_2 vs CDM 3F DFO -H_2O_2, p= 2.770e-01
CDM +H_2O_2 vs CDM DFO -H_2O_2, p= 6.370e-01	CDM 3F +H_2O_2 vs CDM 3F DF0 +H_2O_2, p= 5.270e-01
CDM +H_2O_2 vs CDM 3F -H_2O_2, p= 1.060e-01	CDM DFO- H_2O_2 vs CDM DFO +H_2O_2 p= 1.000e+00
CDM +H_2O_2 vs CDM 3F +H_2O_2, p=9.281e-01	CDM DFO -H_2O_2 vs CDM 3F DFO -H_2O_2, p=6.582e-01
CDM +H_2O_2 vs CDM 3F DFO -H_2O_2, p=1.000e+00	CDM DFO -H_2O_2 vs CDM 3F DF0 +H_2O_2, p=9.995e-01
CDM +H_2O_2 vs CDM 3F DF0 +H_2O_2, p=8.185e-01	CDM DFO +H_2O_2 vs CDM 3F DFO +H_2O_2, p=9.259e-01
CDM DFO +H2O2 vs CDM 3F DFO -H2O2, p=2.260e-01	CDM 3F DFO +H_2O_2 vs CDM 3F DFO -H_2O_2, p=8.843e-01

Table S10. P values for Collagen IV WC-CTCF

D10S -H_2O_2 vs D10S +H_2O_2, p= 9.820e-01	D10S 3F -H_2O_2 vs D10S 3F +H_2O_2, p= 1.000e-05
D10S - H_2O_2 vs D10S DFO +H_2O_2 p= 9.770e-01	D10S 3F - H_2O_2 vs D10S DFO +H_2O_2 p= 4.400e-04
D10S -H_2O_2 vs D10S DFO -H_2O_2, p= 1.000e+00	D10S 3F -H_2O_2 vs D10S DFO -H_2O_2, p= 0.000e+00
D10S -H_2O_2 vs D10S 3F -H_2O_2, p= 2.900e-04	D10S 3F -H_2O_2 vs D10S 3F DFO -H_2O_2, p= 1.000e-05
D10S -H_2O_2 vs D10S 3F +H_2O_2, p= 1.000e+00	D10S 3F -H_2O_2 vs D10S 3F DFO +H_2O_2, p= 2.600e-04
D10S -H_2O_2 vs D10S 3F DFO -H_2O_2, p= 7.750e-01	D10S 3F + H_2O_2 vs D10S DFO +H_2O_2 p= 4.110e-01
D10S -H_2O_2 vs D10S 3F DF0 +H_2O_2, p= 9.420e-01	D10S 3F +H_2O_2 vs D10S DFO -H_2O_2, p= 4.330e-01
D10S + H_2O_2 vs D10S DFO +H_2O_2, p= 1.000e+00	D10S 3F +H_2O_2 vs D10S 3F DFO -H_2O_2, p= 1.680e-01
D10S +H_2O_2 vs D10S DFO -H_2O_2, p= 9.270e-01	D10S 3F +H_2O_2 vs D10S 3F DF0 +H_2O_2, p= 8.690e-01
D10S +H_2O_2 vs D10S 3F -H_2O_2, p= 1.000e-05	D10S DFO- H_2O_2 vs D10S DFO +H_2O_2 p= 8.700e-01
D10S +H_2O_2 vs D10S 3F +H_2O_2, p= 6.150e-01	D10S DFO -H_2O_2 vs D10S 3F DFO -H_2O_2, p= 4.330e-01
D10S +H_2O_2 vs D10S 3F DFO -H_2O_2, p= 9.360e-01	D10S DFO -H_2O_2 vs D10S 3F DFO +H_2O_2, p= 7.930e-01
D10S +H_2O_2 vs D10S 3F DF0 +H_2O_2, p= 1.630e-01	D10S DFO +H_2O_2 vs D10S 3F DFO +H_2O_2, p= 1.000e-02
D10S DFO +H2O2 vs D10S 3F DFO -H2O2, p= 6.650e-01	D10S 3F DFO +H_2O_2 vs D10S 3F DFO -H_2O_2, p= 3.000e-02
CDM -H_2O_2 vs CDM +H_2O_2, p= 1.000e-03	CDM 3F -H_2O_2 vs CDM 3F +H_2O_2, p= 9.640e-01
CDM - H_2O_2 vs CDM DFO +H_2O_2 p= 4.100e-02	CDM 3F - H_2O_2 vs CDM DFO +H_2O_2 p= 5.100e-02
CDM -H_2O_2 vs CDM DFO -H_2O_2, p= 2.100e-02	CDM 3F -H_2O_2 vs CDM DFO -H_2O_2, p= 9.300e-04
CDM -H_2O_2 vs CDM 3F -H_2O_2, p= 1.000e+00	CDM 3F -H_2O_2 vs CDM 3F DFO -H_2O_2, p= 1.420e-01
CDM -H_2O_2 vs CDM 3F +H_2O_2, p= 9.870e-01	CDM 3F -H_2O_2 vs CDM 3F DF0 +H_2O_2, p= 1.600e-02
CDM -H_2O_2 vs CDM 3F DFO -H_2O_2, p= 1.810e-01	CDM 3F + H_2O_2 vs CDM DFO +H_2O_2 p= 3.400e-02
CDM -H_2O_2 vs CDM 3F DFO +H_2O_2, p= 1.060e-01	CDM 3F +H_2O_2 vs CDM DFO -H_2O_2, p= 1.000e-03
CDM + H_2O_2 vs CDM DFO +H_2O_2, p= 6.730e-01	CDM 3F +H_2O_2 vs CDM 3F DFO -H_2O_2, p= 9.500e-02
CDM +H_2O_2 vs CDM DFO -H_2O_2, p= 1.400e-02	CDM 3F +H_2O_2 vs CDM 3F DF0 +H_2O_2, p= 1.100e-02
CDM +H_2O_2 vs CDM 3F -H_2O_2, p= 2.100e-04	CDM DFO- H_2O_2 vs CDM DFO +H_2O_2 p= 8.690e-01
CDM +H_2O_2 vs CDM 3F +H_2O_2, p= 1.100e-04	CDM DFO -H_2O_2 vs CDM 3F DFO -H_2O_2, p=6.582e-01
CDM +H_2O_2 vs CDM 3F DFO -H_2O_2, p= 7.900e-02	CDM DFO -H_2O_2 vs CDM 3F DF0 +H_2O_2, p=9.995e-01
CDM +H_2O_2 vs CDM 3F DF0 +H_2O_2, p= 3.000e-03	CDM DFO +H_2O_2 vs CDM 3F DFO +H_2O_2, p=9.259e-01
CDM DFO +H2O2 vs CDM 3F DFO -H2O2, p= 6.640e-01	CDM 3F DFO +H_2O_2 vs CDM 3F DFO -H_2O_2, p=8.843e-01
